# Upadacitinib for atopic dermatitis induced by secukinumab in patients with refractory ankylosing spondylitis:a case report

**DOI:** 10.3389/fimmu.2026.1737773

**Published:** 2026-05-01

**Authors:** Jinxia Fang, Shaobiao Pan

**Affiliations:** Department of Rheumatology and Immunology, Taizhou Hospital of Zhejiang Province affiliated to Wenzhou Medical University, Taizhou, Zhejiang, China

**Keywords:** ankylosing spondylitis, atopic dermatitis, drug-induced atopic dermatitis, immune deviation, interleukin-17 inhibitor, paradoxical adverse reaction, secukinumab, upadacitinib

## Abstract

Ankylosing spondylitis (AS) is a chronic inflammatory disease that primarily affects the sacroiliac joints, spine, and peripheral joints. The widespread application of biological agents has led to significant clinical improvements in the management of AS. However, some patients may develop paradoxical immune deviation following treatment with biologics, such as eczematous drug-induced rash, drug-related vasculitis, and inflammatory bowel disease (IBD).In this study, we present a case of a 25-year-old female patient diagnosed with AS who developed secondary loss of response following 6 months of adalimumab (ADA) therapy. After completing ADA washout, she was started on secukinumab (SEC) treatment. Subsequent to the fourth dose of SEC, the patient developed worsening low back pain accompanied by a severe eczematous rash. Treatment was then switched to upadacitinib (UPA). After 6 months of UPA administration, both her AS and drug-induced atopic dermatitis (AD) achieved significant clinical remission.

## Introduction

Biological agents targeting interleukin-17 (IL-17) and tumor necrosis factor (TNF) have been widely used in the treatment of AS with well-established efficacy. However, these agents may occasionally induce paradoxical reactions (PRs) in clinical practice, specifically the induction or exacerbation of immune-mediated pathological damage while treating the primary disease. PRs, including paradoxical psoriasis (PP) and paradoxical arthritis, have been reported in the literature, particularly those associated with TNF inhibitors (1). Additionally, several cases of cutaneous PRs related to IL-17 inhibitors have also been documented, including PP,oral ulcers, sarcoidosis-like rashes, alopecia areata, and pyoderma gangrenosum (2, 3). Herein, we report a case of a patient with AS who developed AD following treatment with SEC, and subsequently achieved remission after switching to UPA therapy.

## Case presentation

**Figure 1:**
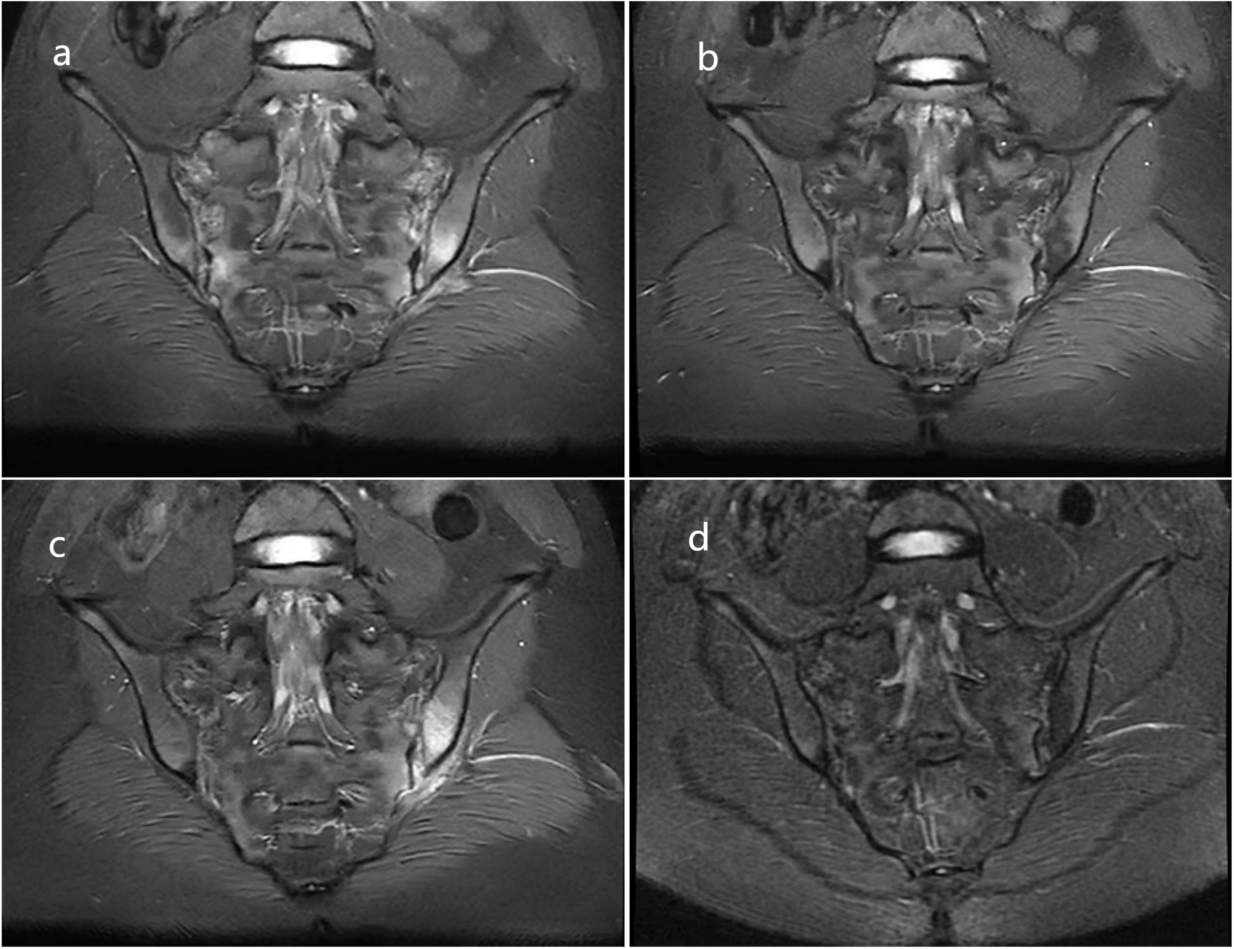
Sacral-iliac joint MRI: Before adalimumab treatment **(A)** and 6 months after adalimumab treatment **(B)**; Before secukinumab and upadacitinib treatment **(C)** and 6 months after upadacitinib treatment **(D)**.

A 25-year-old female patient with a prior history of urticaria presented to our outpatient clinic with a 1-year history of recurrent low back pain and hip pain. Laboratory testing was positive for HLA-B27, and imaging studies confirmed bilateral sacroiliitis. The patient was diagnosed with AS in accordance with the 1984 revised New York criteria for AS (**Figure 1A**).

In October 2023, she was initiated on adalimumab(ADA)40 mg every 2 weeks, which resulted in significant improvement of her back pain. Follow-up magnetic resonance imaging (MRI) of the sacroiliac joints 6 months later revealed marked resolution of inflammation compared with baseline (**Figure 1B**), and the dosing regimen was adjusted to ADA 40 mg once monthly. Nevertheless, recurrent low back pain developed after the 16th administration of ADA, suggestive of secondary loss of response. Repeat MRI confirmed recurrence of sacroiliac joint inflammation (**Figure 1C**).

**Figure 2:**
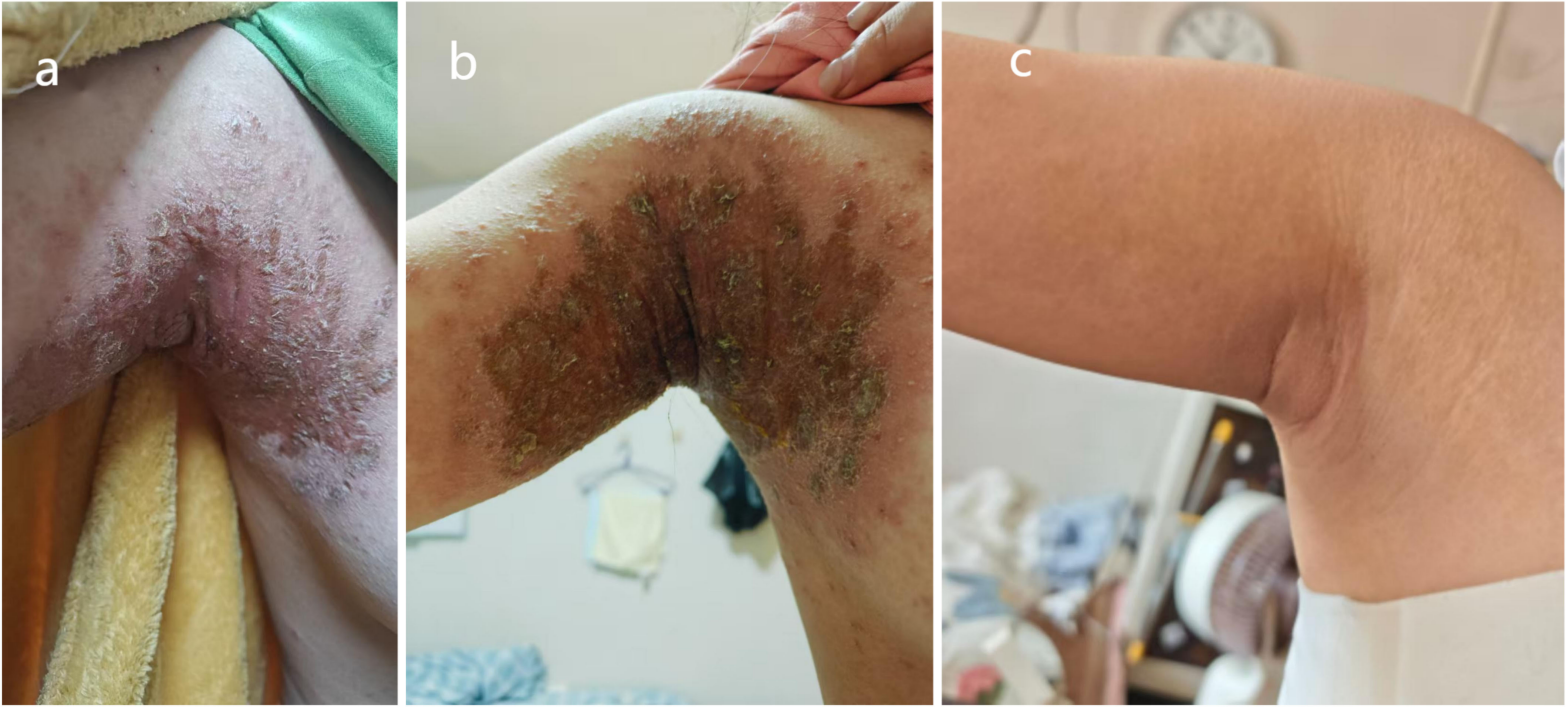
Eczematous rash: comparison at 1 month post-secukinumab initiation **(A, B)** and 6 months post-upadacitinib initiation **(C)**.

Accordingly, after a 4-week washout period following discontinuation of adalimumab, the patient was started on SEC in September 2024. After the fourth dose of SEC, she developed a rash in the axillary region (**Figure 2A**) accompanied by severe pruritus, with negative serum total IgE. A dermatologist confirmed the diagnosis of drug-related AD. Discontinuation of SEC was recommended, combined with topical glucocorticoids and oral loratadine plus cetirizine. However, the rash persisted and progressed in severity (**Figure 2B**).

To simultaneously control both AS and AD, oral UPA (15 mg once daily) was administered. Complete resolution of low back pain and skin rash was achieved after 2 months of treatment (**Figure 2C**), and follow-up MRI at 6 months confirmed complete resolution of sacroiliac joint inflammation (**Figure 1D**). Serum IgE, erythrocyte sedimentation rate, C-reactive protein, and other laboratory parameters remained within normal ranges throughout the treatment period.

## Discussion

As a key bridge between the innate and adaptive immune responses, interleukin-17A (IL-17A) plays a crucial pathogenic role in a variety of immune-mediated diseases, including psoriasis, psoriatic arthritis (PsA), AS, and rheumatoid arthritis (RA) (4). SEC is a selective inhibitor of interleukin-17A (IL-17A) and has been approved for the treatment of autoimmune diseases such as AS and psoriasis. By blocking the interaction between IL-17A and its receptor, this drug can effectively inhibit the release of proinflammatory cytokines (including tumor necrosis factor-α [TNF-α], interferon-γ [IFN-γ], and interleukin-6 [IL-6]) as well as other chemokines—factors that play a crucial role in the pathophysiological process of AS (5). With the widespread application of SEC in real-world clinical practice, an increasing number of SEC-related adverse reactions have been reported, including inflammatory bowel disease (IBD), eczematous drug-induced rash, drug-related vasculitis, drug-induced lupus erythematosus, drug-induced psoriasis and oral mucositis (2, 3).

As of now, more than 30 cases of eczematous drug reactions have been reported in psoriasis patients receiving SEC treatment, including eczematous, AD-like, and psoriasiform rashes (6–9). The histopathological findings in our case series were consistent with the unique clinical features of each patient. In fact, although all lesions shared varying degrees of spongiosis as a common feature, distinct clinical subtypes exhibited characteristic histopathological alterations. Given that the clinical presentation of the present patient was highly consistent with an atopic dermatitis-like eruption, further skin biopsy was not performed.Surprisingly, SEC-related eczematous rashes are relatively uncommon in AS. Studies have shown that eczematous rashes mostly occur 2 to 76 weeks (with an average of 20 weeks) after SEC treatment, and they are mainly moderate to severe in severity. Among these patients, 67% discontinued SEC due to eczematous lesions and initiated treatment with topical or systemic glucocorticoids and cyclosporine (10).

In this case, the patient developed a skin rash within 1 month of SEC administration. No other medications or chemical agents were administered or exposed during treatment, and a clear temporal correlation was observed between drug initiation and rash onset. Therefore, SEC-related dermatitis was regarded as the primary diagnosis. Although SEC therapy is associated with an increased risk of infections, such infections mainly manifest as upper respiratory tract infections and candidal infections, whereas cutaneous candidiasis is relatively rare (11). The typical presentation of cutaneous candidiasis consists of bright red, moist macules with well-defined margins, often surrounded by satellite pustules—findings inconsistent with the rash observed in this case. The patient’s skin rash rapidly improved following UPA therapy, further supporting an immune-related pathogenesis rather than an infectious etiology.

At present, the specific mechanism by which these eczematous drug adverse reactions are induced during anti-IL-17A treatment remains unclear. Such drugs may mainly block the Th1 immune pathway, thereby causing an imbalance in the Th1/Th2 immune response and further promoting the Th2 pathway involved in the pathogenesis of eczema (12). Inhibition of IL-17A may also upregulate the expression of other members of IL-17 family, such as interleukin-17C (IL-17C), thereby driving the inflammatory signal cascade during the development of these rashes. IL-17C can promote T lymphocytes to synthesize interleukin-17A/F (IL-17A/F) and interleukin-22 (IL-22); therefore, blocking IL-17C may be beneficial for both psoriasis and atopic dermatitis (13). Based on this hypothesis, brodalumab— as the only anti-IL-17 drug capable of blocking IL-17A, IL-17F, and IL-17C simultaneously— may be beneficial for patients who develop paradoxical eczematous reactions after using IL-17A antagonists. However, it should be noted that clinical trial data have reported a 4% incidence of mucocutaneous candidiasis. Fortunately, the majority of these cases were mild to moderate in severity, which could be alleviated by topical or oral antifungal therapy and usually did not require drug discontinuation. Paradoxical cutaneous reactions related to brodalumab are rare, with only 3 cases of pustular eruptions having been reported to date (14). Nevertheless, no similar cases have been reported in the current literature, and more clinical experience still needs to be accumulated (10).

In recent decades, the latest research has revealed that many cytokines identified as the fundamental drivers of autoimmune diseases transmit signals through the JAK-STAT (Janus Kinase-Signal Transducer and Activator of Transcription) pathway (15).Upadacitinib is an orally administered, selective, and reversible Janus kinase (JAK) inhibitor that has been approved by the US Food and Drug Administration (FDA), European Medicines Agency (EMA), and other regulatory authorities for the treatment a variety of autoimmune diseases, including active AS and moderate-to-severe AD (16).It has shown satisfactory efficacy in patients with AS who have an inadequate response to biological disease-modifying antirheumatic drugs (bDMARDs), specifically tumor necrosis factor inhibitors or interleukin-17 inhibitors) (17). In the treatment of AD, compared with dupilumab, upadacitinib has demonstrated superior efficacy in patients with moderate-to-severe AD, and no new safety concerns have been identified (18).

To our knowledge, reports regarding the use of UPA for the treatment of SEC-induced AD in patients with AS remain limited, there have been reports of JAK inhibitors successfully treating SEC-related SAPHO syndrome, alopecia areata, and palmoplantar pustulosis (19–21).This means that when drug-related AD occurs in patients with AS, UPA may be a better treatment option. At the same time, we should also note that when SEC is used for treatment, special attention should be paid to the patient’s skin condition.

## Conclusion

This case describes a 25-year-old female patient with AS who had an inadequate response to initial tumor necrosis factor inhibitor (TNFI) treatment. After receiving SEC therapy, she developed a severe rash, which was considered drug-related AD. Subsequent treatment with UPA effectively controlled her symptoms. This also demonstrates the great potential of UPA in managing IL-17-related rashes.

## Data Availability

The raw data supporting the conclusions of this article will be made available by the authors, without undue reservation.
